# Immune cell isolation from lymphoid and nonlymphoid organs

**DOI:** 10.1016/j.mocell.2025.100277

**Published:** 2025-09-19

**Authors:** Jong Seok Park, Yoontae Lee

**Affiliations:** Department of Life Sciences, Pohang University of Science and Technology (POSTECH), Pohang, Gyeongbuk, Republic of Korea

**Keywords:** Bone marrow, Kidney, Lymph node, Peritoneal cavity, Spleen

## Abstract

Immune cells are distributed across various tissues. While a majority are concentrated in primary and secondary lymphoid organs such as the bone marrow, thymus, lymph nodes, and spleen, a subset resides in nonlymphoid organs, including the kidney, liver, and lung, as well as the peritoneal cavity, where they play critical roles in local immune surveillance and response. In this editorial, we outline concise and practical protocols for the isolation of immune cells from a range of lymphoid and nonlymphoid organs.

## INTRODUCTION

Immune cells are primarily distributed in the lymphoid organs, including the bone marrow, thymus, spleen, tonsils, and lymph nodes. Recent studies have demonstrated their presence in nonlymphoid organs, where they contribute to the local immune defense against pathogen invasion (Li et al., 2025). The localization and function of immune cells in different organs are significantly influenced by their specific microenvironments or tissue niches. Therefore, isolation techniques tailored to each organ are critical for accurate immunological analysis. Inadequate or inappropriate cell preparation can lead to contamination and misinterpretation of experimental data. Here, we present an overview of the standardized methods for isolating immune cells from both lymphoid and nonlymphoid organs.

## IMMUNE CELL ISOLATION FROM LYMPHOID ORGANS

**Fig. 1:**
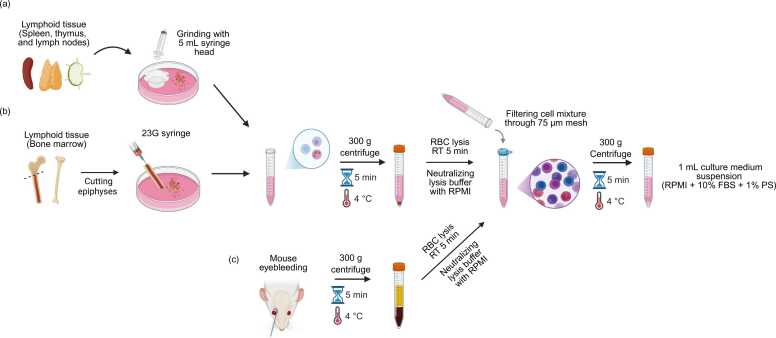
Schematic overview of immune cell isolation from lymphoid and blood compartments. (a) Immune cell isolation from dense lymphoid tissues. (b) Immune cell isolation from bone marrow. (c) Immune cell isolation from peripheral blood. Created in BioRender.

Dense lymphoid tissues, such as the thymus, spleen, and lymph nodes, are mechanically dissociated using the plunger end of a syringe to generate a single-cell suspension in a 100-mm tissue culture dish containing medium (Harada et al., 2022). The resulting cell suspension is filtered through a 75-μm mesh into a 15-ml conical tube to remove debris. Subsequently, the filtered cells are centrifuged at 300*g* for 5 minutes at 4°C to collect the cell pellet. Thymus and lymph node tissues generally lack red blood cells (RBCs); therefore, the resulting pellets can be directly resuspended in 1 ml of culture medium composed of RPMI (Roswell Park Memorial Institute)-1640, 10% fetal bovine serum, and 1% penicillin-streptomycin (Kim et al., 2021). In contrast, the spleen contains a large number of RBCs that can impede accurate cell counting and analysis. To address this, RBCs are lysed using a RBC lysis buffer composed of 155 mM NH₄Cl, 12 mM KHCO₃, and 0.1 mM EDTA (Park et al., 2024a, Park et al., 2024b). The high salt concentration in this buffer selectively lyses erythrocytes following a 5-minute incubation at room temperature. The lysis reaction is then neutralized by adding 5 ml of PRMI-1640 medium, followed by filtration through a 75-μm mesh to remove residual debris generated during lysis. The filtered cell mixture is centrifuged again under the same conditions as those described above to obtain clean cell pellets (Fig. 1a).

For isolation from bone marrow, the femur and tibia are dissected by removing the surrounding muscle tissue using a pair of scissors and separated by gently twisting the bones at the joint. The epiphyses are cut to expose the marrow cavity. A 23-gauge needle attached to a syringe filled with RPMI-1640 medium is then inserted into the exposed bone ends to flush out bone marrow cells. This flushing process is repeated at least twice to maximize the yield, and gentle scraping with a needle tip further enhances the cell yield (Toda et al., 2021). Bone marrow contains erythroid progenitors; therefore, an additional RBC lysis step, similar to that used for spleen, is required to isolate immune cells (Fig. 1b). Immune cell yields from the major immune organs of adult mice are presented in Table 1.

**Table 1:** Immune cell yields and viability after single-cell isolation

Organ	Viability	Immune cell yield	Reference
Thymus	∼95%	∼100 × 10^6^	Theofilatos et al., 2022, Xiao et al., 2003
Spleen	∼95%	∼80 × 10^6^	Look et al. (2023)
Lymph node	∼95%	∼2 × 10^6^	Menzel et al. (2022)
Bone marrow	∼95%	∼50 × 10^6^	Colvin et al. (2004)
Lung	∼85%	∼1.5 × 10^6^	Sauer et al. (2006)
Liver	∼95%	∼15 × 10^6^	Blom et al., 2009, Li et al., 2023
Kidney	∼95%	∼1.4 × 10^6^	Ascon et al., 2008, Nistala et al., 2016

Blood collected via retro-orbital bleeding or cardiac puncture is immediately transferred to tubes containing 1 mM EDTA buffer (Prepared in phosphate-buffered saline) (Efthymiou et al., 2022). The RBCs are subsequently lysed using a lysis buffer (Horn et al., 2008) (Fig. 1c).

## IMMUNE CELL ISOLATION FROM NONLYMPHOID TISSUES/ORGANS

**Fig. 2:**
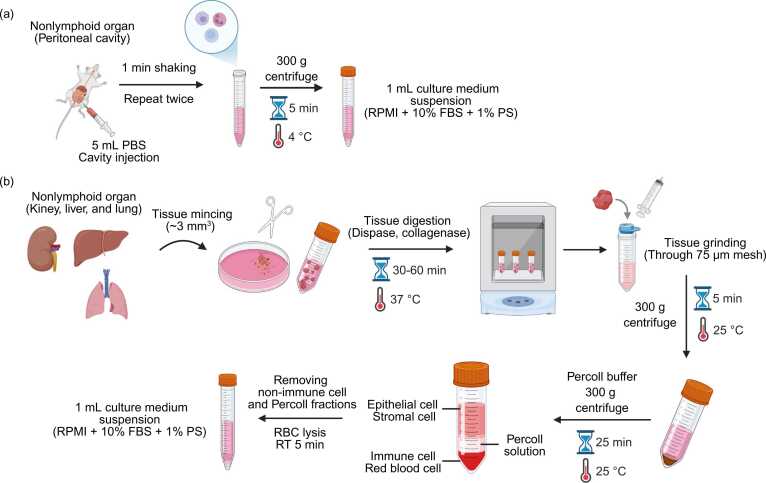
Schematic overview of immune cell isolation from nonlymphoid organs. (a) Immune cell isolation from the peritoneal cavity. (b) Immune cell isolation from solid organs (liver, lung, and kidney). Created in BioRender.

Immune cells from the peritoneal cavity are collected by carefully injecting 5 ml of PBS into the cavity using a 23-gauge needle, ensuring not to puncture the peritoneal wall. The abdominal area is gently agitated for approximately 1 minute, after which PBS is aspirated using the same syringe. This process is typically repeated at least twice to increase cell yield (Hong et al., 2022, Ray and Dittel, 2010). Care must be taken to avoid puncturing the blood vessels, as this can lead to contamination by peripheral blood cells (Fig. 2a).

Owing to their dense fibrous structure, the liver and lung tissues cannot be dissociated by mechanical disruption alone. Therefore, enzymatic digestion is required to ensure the efficient isolation of immune cells. Digestive enzymes such as collagenases and dispases are commonly used to degrade fibrous tissue regions. As collagenases exhibit tissue-specific activity, selecting an appropriate enzyme formulation based on the target organ is essential (D'Agostino et al., 2022, Lemaitre et al., 2024, Liao et al., 2020, Zhou et al., 2023). Detailed organ-specific digestion buffer compositions are provided in Table 2. To facilitate digestion, tissues are first minced into small fragments (∼3 mm^3^) and then incubated in an enzyme-containing medium for 30 to 60 minutes at 37°C in a shaking incubator. Once the tissues soften, they are further dissociated using the plunger end of the syringe. Following mechanical dissociation, the cell suspensions are filtered through a 75-μm mesh to remove undigested debris and then centrifuged at 300*g* to collect the cells. The resulting pellet contains a mixture of immune and tissue-derived stromal cells. To purify the immune cells, the cell suspension is resuspended in Percoll solution (prepared in PBS or PRMI-1640) and subjected to density gradient centrifugation (D'Agostino et al., 2022, Lemaitre et al., 2024, Liao et al., 2020, Prosser et al., 2021, Zhou et al., 2023). Percoll solution concentrations may vary depending on tissue type (Table 2). To preserve the integrity of the gradient, centrifugation should be performed at 300*g* without the use of the deceleration brake for 25 minutes at 25°C. After centrifugation, 2 distinct layers are formed: the upper layer contains stromal cells and the lower layer is enriched with immune cells. The upper layer and supernatant are carefully removed to isolate the immune cells and RBC fractions from the liver and lung tissues (Fig. 2b). The RBCs are subsequently lysed using a lysis buffer to obtain purified immune cells. Immune cell yields from nonlymphoid organs in adult mice are presented in Table 1.

**Table 2:** Composition of digestion buffers used for immune cell isolation from nonlymphoid organs

Reagent	Final concentration	Reference
*Lung digestion solution*		
RPMI-1640	N/A	D'Agostino et al., 2022, Lemaitre et al., 2024
FBS	10%	
HEPES (optional)	10 mM	
Penicillin (optional)	100 U/ml	
Streptomycin (optional)	100 mg/ml	
Collagenase type 1	150 U/ml	
37°C shaking incubator at 225 rpm for 60 min		
40% Percoll/PBS or RPMI buffer (40% Percoll + 60% PBS or RPMI) for gradient centrifugation		

*Liver digestion solution*		
DMEM	N/A	Prosser et al., 2021, Zhou et al., 2023
Collagenase type 2	2 mg/ml	
37°C shaking incubator at 80-100 rpm for 30 min		
42% Percoll/PBS or RPMI buffer (42% Percoll + 58% PBS or RPMI) for gradient centrifugation		

*Kidney digestion solution*		
RPMI-1640	N/A	Liao et al. (2020)
FCS	2%	
Collagenase type B	1 mg/ml	
37°C shaking incubator at 60 rpm for 45 min		
44% Percoll/PBS or RPMI buffer (44% Percoll + 56% PBS or RPMI) for gradient centrifugation		

## TROUBLESHOOTING

If the initial filtration through a 75-μm mesh is incomplete, 2 meshes can be used in combination. During gradient centrifugation, distinct cell layers are formed; the upper stromal cell layer should be carefully removed by suction. As these layers can be easily disrupted by mechanical disturbance, removal should proceed sequentially from the top rather than the intermediate fraction. This prevents contamination of the immune cell pellet with debris and enables the isolation of highly purified immune cells.

Inappropriate enzyme selection or prolonged enzymatic treatment may alter the expression of surface proteins, including key receptors, thereby compromising downstream analyses (Abuzakouk et al., 1996, Autengruber et al., 2012). For example, treatment with collagenase F has no significant effect on dendritic cells (DCs), whereas that with collagenase type IV causes a broad loss of DC marker expression in skin-derived DCs (Botting et al., 2017). In a previous study, collagenase treatment resulted in an increase in overall cell yield with longer incubation; the expression of cell-surface markers markedly reduced at 90 minutes, which affected flow cytometry gating (Chen et al., 2014). These findings highlight that both enzyme choice and incubation duration critically influence the quality of immune cell isolation from different organs.

## CONCLUDING REMARKS

Given the distinct structural characteristics and immune cell distribution across tissues, organ-specific approaches are essential for accurate analysis. Minimizing the time required for immune cell isolation is critical because prolonged ex vivo exposure can compromise both cell viability and functionality. This is particularly important for fibrous organs, where enzymatic digestion is necessary to disrupt the tissue matrix. Extended enzymatic treatment may alter the expression of surface proteins, including key receptors, thereby affecting downstream analyses. Therefore, rapid and optimized processing is essential to preserve the natural state of immune cells for reliable experimental outcomes.

## Author Contributions

**Yoontae Lee:** Writing – review & editing. **Jong Seok Park:** Writing – original draft, Methodology, Conceptualization.

## Declaration of Competing Interests

The authors declare no conflicts of interest.

## References

[bib1] Abuzakouk M., Feighery C., O'Farrelly C. (1996). Collagenase and dispase enzymes disrupt lymphocyte surface molecules. J. Immunol. Methods.

[bib2] Ascon D.B., Ascon M., Satpute S., Lopez-Briones S., Racusen L., Colvin R.B., Soloski M.J., Rabb H. (2008). Normal mouse kidneys contain activated and CD3+CD4- CD8- double-negative T lymphocytes with a distinct TCR repertoire. J. Leukoc. Biol..

[bib3] Autengruber A., Gereke M., Hansen G., Hennig C., Bruder D. (2012). Impact of enzymatic tissue disintegration on the level of surface molecule expression and immune cell function. Eur. J. Microbiol. Immunol. (Bp).

[bib4] Blom K.G., Qazi M.R., Matos J.B., Nelson B.D., DePierre J.W., Abedi-Valugerdi M. (2009). Isolation of murine intrahepatic immune cells employing a modified procedure for mechanical disruption and functional characterization of the B, T and natural killer T cells obtained. Clin. Exp. Immunol..

[bib5] Botting R.A., Bertram K.M., Baharlou H., Sandgren K.J., Fletcher J., Rhodes J.W., Rana H., Plasto T.M., Wang X.M., Lim J.J.K. (2017). Phenotypic and functional consequences of different isolation protocols on skin mononuclear phagocytes. J. Leukoc. Biol..

[bib6] Chen Z., Chen X., Xu Y., Xiong P., Fang M., Tan Z., Gong F., Zheng F. (2014). Collagenase digestion down-regulates the density of CD27 on lymphocytes. J. Immunol. Methods.

[bib7] Colvin G.A., Lambert J.F., Abedi M., Hsieh C.C., Carlson J.E., Stewart F.M., Quesenberry P.J. (2004). Murine marrow cellularity and the concept of stem cell competition: geographic and quantitative determinants in stem cell biology. Leukemia.

[bib8] D'Agostino M.R., Afkhami S., Kang A., Marzok A., Miller M.S., Xing Z. (2022). Protocol for isolation and characterization of lung tissue resident memory T cells and airway trained innate immunity after intranasal vaccination in mice. STAR Protoc..

[bib9] Efthymiou A., Mureanu N., Pemberton R., Tai-MacArthur S., Mastronicola D., Scottà C., Lombardi G., Nicolaides K.H., Shangaris P. (2022). Isolation and freezing of human peripheral blood mononuclear cells from pregnant patients. STAR Protoc..

[bib10] Harada Y., Miyamoto K., Sujino T. (2022). Protocol to isolate and enrich mouse splenic naive CD4(+) T cells for in vitro CD4(+)CD8αα(+) cell induction. STAR Protoc..

[bib11] Hong H., Lee J., Park G.Y., Kim S., Park J., Park J.S., Song Y., Lee S., Kim T.J., Lee Y.J. (2022). Postnatal regulation of B-1a cell development and survival by the CIC-PER2-BHLHE41 axis. Cell Rep..

[bib12] Horn P., Bork S., Diehlmann A., Walenda T., Eckstein V., Ho A.D., Wagner W. (2008). Isolation of human mesenchymal stromal cells is more efficient by red blood cell lysis. Cytotherapy.

[bib13] Kim S., Park G.Y., Park J.S., Park J., Hong H., Lee Y. (2021). Regulation of positive and negative selection and TCR signaling during thymic T cell development by capicua. Elife.

[bib14] Lemaitre P., Mathieu C., Gysemans C. (2024). Protocol for murine multi-tissue deep immunophenotyping using a 40-color full-spectrum flow cytometry panel. STAR Protoc..

[bib15] Li J., Xiao C., Li C., He J. (2025). Tissue-resident immune cells: from defining characteristics to roles in diseases. Signal Transduct. Target. Ther..

[bib16] Li Y.T., Wu H.L., Wu L.L. (2023). Isolation of murine hepatic myeloid cells with high yield and purity using immunomagnetic beads for subset analysis. STAR Protoc..

[bib17] Liao W., Ma C., Zhang N. (2020). Isolation of mouse kidney-resident CD8+ T cells for flow cytometry analysis. J. Vis. Exp..

[bib18] Look T., Meister H., Weller M., Weiss T. (2023). Protocol for the expansion of mouse immune effector cells for in vitro and in vivo studies. STAR Protoc..

[bib19] Menzel L., Zschummel M., Rehm A. (2022). Analyses of murine lymph node endothelial cell subsets using single-cell RNA sequencing and spectral flow cytometry. STAR Protoc..

[bib20] Nistala R., Meuth A., Smith C., Annayya A. (2016). Reliable and high efficiency extraction of kidney immune cells. J. Vis. Exp..

[bib21] Park J., Lee J., Hur Y., Kim C.J., Kim H.B., Um D., Kim D.S., Lee J.Y., Park S., Park Y. (2024). ETV5 promotes lupus pathogenesis and follicular helper T cell differentiation by inducing osteopontin expression. Proc. Natl. Acad. Sci. U. S. A..

[bib22] Park J.S., Kang M., Kim H.B., Hong H., Lee J., Song Y., Hur Y., Kim S., Kim T.K., Lee Y. (2024). The capicua-ataxin-1-like complex regulates Notch-driven marginal zone B cell development and sepsis progression. Nat. Commun..

[bib23] Prosser A., Dart S., Larma-Cornwall I., Lucas M. (2021). Flow cytometric characterization of tissue-resident lymphocytes after murine liver and heart transplantation. STAR Protoc..

[bib24] Ray A., Dittel B.N. (2010). Isolation of mouse peritoneal cavity cells. J. Vis. Exp..

[bib25] Sauer K.A., Scholtes P., Karwot R., Finotto S. (2006). Isolation of CD4+ T cells from murine lungs: a method to analyze ongoing immune responses in the lung. Nat. Protoc..

[bib26] Theofilatos D., Äijö T., Tsagaratou A. (2022). Protocol to isolate mature thymic T cell subsets using fluorescence-activated cell sorting for assessing gene expression by RNA-seq and transcription factor binding across the genome by CUT&RUN. STAR Protoc..

[bib27] Toda G., Yamauchi T., Kadowaki T., Ueki K. (2021). Preparation and culture of bone marrow-derived macrophages from mice for functional analysis. STAR Protoc..

[bib28] Xiao S.Y., Li Y., Chen W.F. (2003). Kinetics of thymocyte developmental process in fetal and neonatal mice. Cell Res..

[bib29] Zhou X., Meng M., Wu Y., Gao R., Shan Y., Gu S., He J. (2023). Protocol to dissociate and isolate wide-diversity single cells by density gradient centrifugation from human hepatoblastoma tissue. STAR Protoc..

